# Eye Health, COVID-19, and the Occupational Health Professional: Round Table

**DOI:** 10.1177/21650799211022990

**Published:** 2021-07-19

**Authors:** Susan Gallagher, Jay Clasing, Edward Hall, Stephanie Hammond, Gayle Howard, Todd Mohrmann, Jayme Taormina Vaccaro

**Affiliations:** 1 Celebration Institute, Inc.; 2 U.S. Army Public Health Center; 3 Keck Medicine of USC; 4 School of Nursing, The University of Alabama at Birmingham (UAB); 5 Eye Physicians Medical/Surgical Center, Inc.; 6 Dynamic Training; 7 Palo Alto Foundation Medical Group

**Keywords:** eye health, Computer Vision Syndrome, COVID-19, mask wearing

## Abstract

**Background:**

Eye health has garnered increased attention since the COVID-19 pandemic. This Round Table explored the impact mask wearing, delays in eye examinations, and increased screen time have on vision and ultimately the worker.

**Methods:**

Leading experts in the areas of occupational health, risk management, eye health, and communication were identified and invited to participate in a Round Table discussion. Questions posed to experts were based on literature that addressed eye health, such as mask wearing, communication and managing expectations when accessing professional eye health appointments, and increased screen time.

**Findings:**

Experts agreed that eye health considerations must be in place. These considerations should address not only clinical care of the patient but ways to protect workers from occupational injury associated with the eye.

**Conclusion/Application to practice:**

The occupational health professional is a key resource for assessment and training that pertains to eye health.

## Background

Most Americans, and therefore most workers, take eye health for granted. Conditions associated with the COVID-19 pandemic have led to an increased awareness of eye health. Dry eyes may occur due to prolonged mask wearing. Wearing a mask or eyewear that is not centered properly because the eyewear is competing with space on the bridge of the nose or fogging lenses may contribute to slips, trips, and falls in the workplace (Kal et al., 2020; Malik & Malik, 2011). Visual barriers may lead to balance issues that further place the worker at risk for slips, trips, or falls (Redfern et al., 2001). Workers explain they are hesitant to make and keep eye health appointments because there is uncertainty about office precautions. Prolonged screen time among workers who previously conducted in-person meetings has had an impact on general health, but the eyes in particular (American Optometric Association [AOA], 2020).

Early in the pandemic, Moshirfar (2020) described mask-associated ocular dryness and irritation in all mask wearers, encouraging healthcare providers to be aware of these threats to eye health. Lubricant eye drops and eye protection, such as goggles, are considered an option that could be used in conjunction with facial masks. Additional care and screening are considerations when working with individuals using masks for extended periods and for workers with a prior history of dry eye disease, recent ophthalmic surgery, or other surface inflammatory diseases, such as Sjogren’s syndrome (Moshirfar et al., 2020).

Sjogren’s syndrome is a common autoimmune disorder that affects 3 million Americans living in the United States, annually. Typically, patients are female between 35 and 50 years old, and have a family history of Sjogren’s syndrome (National Institutes of Health [NIH], 2021). Patients with this disorder experience lacrimal and salivary gland dysfunction, resulting in dry eyes and mouth. They frequently have disruption of taste and smell (Fox & Baer, 2020). No research identified a direct relationship between the threat of mask wearing and the baseline condition of dry eyes associated with Sjogren’s syndrome; however, Moshirfar (2020) specifically recommended additional ophthalmic screening if this condition exists.

Although issues of mask wearing and access to eye care are important, the COVID-19 pandemic has created a more important eye health consideration: Computer Vision Syndrome (CVS). The CVS is also referred to as digital eye strain. The America Optometric Association (AOA, 2020) describes CVS as a group of eye- and vision-related problems that result from prolonged computer, tablet, e-reader, and cell phone use. The CVS is not a new phenomenon. As far back as 2013, experts described CVS and explained that common symptoms of CVS include headaches, blurred vision, dry eyes, neck, and shoulder pain (Reddy et al., 2013).

Solinsky (2020) explains that moving out of properly-lit classrooms and offices, and away from ergonomically designed desks has long-term effects on the eyes. Excessive time at a computer screen under the best conditions can lead to a form of eye strain described earlier; however, the AOA (2020) is especially concerned about this condition during the COVID-19 pandemic.

The Stanford Institute for Economic Policy Research (2020) reports that in 2020, nearly 42% of Americans were working from home and reported spending more time than usual at a computer screen. The Institute reported that when workers spend prolonged time on computer screens, binocular vision and tear film are affected.

Binocular vision helps the eyes converge so a person can see images and words on the screen. The tear film is a thin fluid layer covering the eye that protects the eye and maintains normal function (Dartt & Willcox, 2013). Blinking smooths the surface of the tear film (Braun et al., 2015). Increased use of electronic devices reduces the efficiency of the eye muscles, which means the eyes are less able to converge up close so workers are able to see the computer screen clearly. Solinsky (2017) explains that the tear film function is altered when devices are used excessively because users blink less when looking at screens, which ultimately affects the worker’s ability to focus on the screen.

As the COVID-19 pandemic and its associated restrictions emerge and evolve, mask wearing and access to eye health professionals, and time using screens are also evolving. It is difficult to predict what period of time and to what extent this will continue as a result of COVID-19 precautions. However, experts predict that numerous factors will continue to impact the eye and ultimately vision. This Round Table explores the impact mask wearing, delays in eye examinations, and increased screen time have on vision and ultimately the worker.

## Methods

Experts selected to participate in this Round Table bring a wide variety of medical, risk management, behavior change/communication, and occupational health experience. Representatives from the university setting, insurance industry, research, and healthcare sectors were included. Ophthalmologists and other eye care professionals understand challenges associated with protecting, not only patients but staff members from COVID-19 transmission while delivering eye care in close quarters. Risk managers and insurance analysts are charged with identifying data analytics and targeting risk mitigation from a policy perspective because understanding emerging trends related to the COVID-19 pandemic during and following the pandemic is key to managing risk. Professional educators/trainers understand the value of communication necessary to manage conversations pertaining to transmission prevention. An ophthalmologist, risk managers/analysts, a professional health educator/trainer, researcher, and an occupational health professional were invited to participate.

## Findings

**Masks will likely be a mainstay in transmission precautions. Individuals who use eyewear and masks concurrently explain that visual barriers can occur. What consequences have you seen related to these visual barriers? What are some strategies to overcome these visual barriers?**

### Edward Hall (EH)

Employees, especially early in the pandemic, reported a period of adjustment to masks and face shields. As this safety requirement has been in place for over a year, most of our employees have made an adjustment to this change in practice. However, there are some employees who report visual barriers and we advise these employees to contact an eye professional to screen for exacerbating conditions.

### Stephanie Hammond (SH)

The consequences I have seen wearing eyewear and masks concurrently is fogging of the glasses. Some strategies include a nose bridge in the mask to ensure a better fit, or using medical tape or another tape that is sensitive to the skin and tape down the bridge of the nose to prevent the warm air from escaping.

**Has your organization experienced more fall-related injuries since the COVID-19 pandemic? Is this occurring among all workers? Is the risk for fall-related injury more prevalent among older workers?**

### SH

Our organization has consistently evaluated injuries related to falls during the COVID-19 pandemic among workers. After assessing the workers who had fallen, it was determined the falls were not related to masks or vision issues because of wearing glasses and a mask. The falls were related to workers tripping or slipping on uneven surfaces, water, or ice, or missing a step. According to an article by Kal et al. (2020), it is better to slow down and look ahead, rather than look down while walking with a mask. Looking down reduces stability and this could potentially cause a fall.

### EH

It is too early to have reliable data that tell us whether there has been an increased rate of falls among employees at either the university or the medical centers. However, we make the assumption that employees who wear both prescriptive lenses and masks have more trouble with sight and therefore are at greater risk for falls, regardless of age. However, older workers or workers with issues of balance either because of body weight or aging eyes are encouraged to pursue alternative styles of masks and to regularly use defogging on their glasses.

### SH

Ed, thank you for mentioning body weight. Let me share that an observation from our clinic was that obesity was more of a risk factor for slips, trips, and falls than age.

**Many workers describe a hesitancy in scheduling in-person eye examinations. What are your thoughts about precautions that should be in place?**

### Gayle Howard (GH)

When the pandemic began in our area, our ophthalmology clinic closed except for emergencies and urgent procedures. Initially, our primary concern was protecting our employees from the risk of exposure from patients. Our entire office had a meeting to discuss current recommendations, sick policies, plans for patients, rescheduling, and more. I believe the success of our near shutdown and gradual reopening came from the united efforts of our leaders and staff. Everyone had a say with their concerns, their ideas, and anticipated issues. I believe that has helped our employees to know that we value their health and work very much and that we will support them if a situation evolves where they feel uncomfortable and need our help. If an employee is facing a difficult patient where our guidelines do not seem to be followed, they know that they can engage one of the leaders, whether tech supervisor, manager, optometrist, or medical doctor to support them; step in if necessary; and resolve the situation to keep our office, staff, and patients safe.

As far as actual precautions, all of the leaders in our practice actively review current recommendations and search for additions and changes that we feel make our office safe. We have added spacing in common areas, signage, barriers, and more. In the exam rooms, our examination equipment has much larger barriers separating our face from the patient’s face. We removed paper trays on our slit lamps and clean everything between patients. Rooms are flagged with different colors at different stages of patient care to be sure of clean rooms for the next patient. We limit patient movement around the clinic, limit extra people who accompany patients, and screen patients days prior to and the day of their appointment. We instituted Telehealth for many types of visits and have spread out our schedules to have more days available but with fewer patients per day to allow distancing and thorough cleaning. Employees and patients have commented about how pleased they are with the precautions we have in place.

### Todd Mohrmann (TM)

Eye clinics should strive to create an atmosphere in which any client, a healthcare worker or otherwise, feels confident in the clinic’s commitment to COVID-19 safety protocols and will feel comfortable inquiring about precautions.

Patients will make their first inferences, during scheduling, about a clinic’s level of safety precautions. In my experience, these first interactions are often in need of significant improvement in many healthcare clinics. Clinics should ask themselves:

- How well do scheduling staff communicate about COVID-19 safety?
- Is the communication clear and thorough?
- Do staff sound like they are merely reading a script?
- Do staff ask the patient, while scheduling the appointment, whether they have any safety-related concerns?
- Does the staff communication contain reassuring comments about keeping patients safe during their visit? Do staff sound genuine?

If patients are scheduling appointments via a website or patient portal, how prominent are the clinic’s COVID-19 protocols on the site? Is there an opportunity to submit any safety-related questions via email or the patient portal? Attending to the above considerations can be challenging, but it is time well spent. First impressions are powerful and deserve focused attention.

**Some workers who require eye exams express reluctance to make eye care appointments because of the fear of exposure. What are some ways to prepare patient/workers for a safe environment?**

### TM

Once a patient arrives for an appointment, the safety-related protocols need to once again be prominently on display. Are signs regarding proper masking readily apparent? Is a fully functioning hand sanitizer dispenser available at the entrance, and do front desk staff ask patients to use it? Staff communication upon arrival and throughout the visit will either support or instill doubt regarding the clinic’s safety commitment.

In terms of the clinicians performing the exams, I think clinics should have a standardized protocol that entails clinicians reviewing with all clients the COVID-19 related safety precautions before the examination commences. The protocol should include the clinician asking whether there are any safety concerns and instructing the patient to speak up at any point should concerns arise. A couple of “check-ins” with the patient throughout the encounter should also be part of the clinician’s protocol.

In creating the atmosphere of comfort, I believe nothing is more important than the verbal communication strategies I have described above. That being said, facilities might augment their verbal communication with other strategies, for example, the use of buttons or posters. Several years ago, many hospitals embarked on campaigns to increase staff members’ hand hygiene compliance. This entailed, among other things, having frontline healthcare workers don buttons that say, “Ask me if I washed my hands.” I think eye clinics might consider a similar strategy—buttons worn by clinicians that say, “Ask me about our COVID precautions” or “Ask me how we keep you safe.” Signs with similar messages in every examination room would also be a good strategy. Verbal communication will always be the top priority, but the environment should send the same powerful messages to patients.

**Are there ways patients or workers can initiate conversations when they feel proper precautions are not in place?**

### TM

Despite a clinic’s best efforts in conveying an atmosphere of comfort and confidence, there may still be instances in which patients experience safety-related concerns and are hesitant to speak up. When patients find themselves in these situations during eye health exams, helpful phrases they might use include “I noticed” and “I see that.” For example, “I noticed that you left the room and did not clean your hands again upon re-entering” or “I see that your mask seems to be frequently slipping below your nose.” Patients may feel somewhat more comfortable initiating the conversation by using these phrases. In both instances, the communication that follows the phrase is entirely factual and as such is less likely to provoke a defensive response. If necessary, the patient could follow up by saying, “I’m feeling a little anxious now as a result.” Sharing one’s feelings is a good follow-up strategy because one’s feelings cannot be questioned. The statements could also be reversed, depending on the speaker’s comfort level (i.e., “I’m feeling a little anxious now. I see that your mask seems to be frequently slipping below your nose”). There are a variety of ways to get the message across and clients can choose the approach that is most consistent with the situation and their personal communication style.

**What are some ways the occupational health nurse can track screen time/blue light damage among remote employees? Have policy recommendations been made to workers to prevent this damage? How receptive are workers to recommendations? What about training basic precautions?**

### Jayme Vaccaro (JV)

Even prior to the age of COVID, eye stress due to computer screen exposure was a growing concern. With COVID and working from home, we are potentially not having breaks from the computer that we may have in an office. In addition, with more time at home, we are naturally using our “gadgets” for leisure which further increases our exposure. Test yourself. Compare your screen time now as compared with pre-COVID. Precautions should be taken. Our gadgets track screen time. The information may be at your fingertips. Employers would do well to provide workers with tools to lessen what we don’t want to be the “carpal tunnel” occupational health claim of our generation.

### Jay Clasing (JC)

Remote tracking of screen time/blue light exposure of employees is a challenging proposition. One approach would be to install a retina or facial tracker that is associated with the user being exposed to their display. When a retina or face is recognized, a timer would track total time of exposure. Unfortunately, resistance to this type of technology would most likely be overwhelming. Self-reporting measures are also an option but is generally unreliable.

While there are no specific policies regarding screen time/blue light exposure within my organization, each employee has received education on ways to minimize blue light exposure and eye strain due to prolonged use of digital displays. Our general recommendation is to take a break every 20 minutes and focus on an object at least 20 feet away for 20 seconds (20–20–20 rule). We have also created a blue light tri-fold brochure to discuss the topic and offer recommendations.

Anecdotally, our employees, who are public health personnel, are receptive to these recommendations.

### GH

We have quite a few patients who tell us they are working remotely at this point. They often ask us about their eyes and whether this is damaging to them. There are some conditions which have become more prevalent now, such as dry eye, that are likely being brought on or exacerbated by long periods of screen time. It has always been the recommendation to put in place a few elements to protect your eyes with extended screen time, such as additional lubrication of the eyes with artificial tears, filters to help with blue light and glare, correct prescription glasses for computer/laptop distances of viewing, and planned breaks to allow for more normal blinking of the eyes. While there are programs for employers to monitor screen time of employees, planned breaks and self-monitoring are probably most useful. At this time, there are no real tests for blue light damage to the eye; however, there are concerns that blue light exposure may play a role in age-related changes in the retina, and both tinted and clear blue light filtering glasses are easily available with and without prescription. Use of tears, filters, and good lighting are all fairly easy for employees to institute, but the most common issue is just the constancy of attention to screens. The recommendation is that after 20 minutes of screen time, a break should occur for 20 seconds minimum where the person looks at a distance 20 feet away (and not at another screen). This is the 20–20–20 recommendation. I usually say try and get up and walk around, do something non-screen related, then return to your screen after that short break.

### EH

Many of our employees at the hospital and university are back to the work setting at least some of the time. We have not implemented a policy, but we do train the 20–20–20 recommendation and I think most workers are receptive to this recommendation. A number of studies suggest that employees and student are much more productive when they can avoid CVS and this is motivating. And in addition to training on CVS, we also recognize the health risk associated with sitting that often accompanies increased screen time. Breaks from sitting, adjustable height walking desks, and increased awareness of the risks are one way occupational health professionals can direct employees toward a healthier adjustment to the changing work environment.

**What precautions do you make to patients who develop eye conditions related to a COVID-19, or conditions associated with mask wearing? Do you see more patients with dry eye or pink eye? What about application of eye makeup and eye health in the presence of mask wearing?**

### GH

Masks have definitely contributed to some new issues in ophthalmology. Masks that are ill-fitting and allow air we breathe out to escape upward toward the eyes are associated with dry eye symptoms, such as burning, itching, watering, redness, and blurriness. The humidity of that air often causes fogging of prescription glasses. In addition, masks can ride up and have the impact of decreasing a person’s lower field of vision contributing to increased risk of falls. Using a well-fitting mask, adding a tissue under the upper border, and using special liquids on glasses designed to limit fogging can help with these issues.

Pink eye or conjunctivitis has been seen with COVID-19 with a reported incidence varying from 1.1% in a large U.S. study to as high as 10% in other populations. Luckily, conjunctivitis is usually a self-limited problem with rarely any lasting issues. Another issue has been a focus on eye makeup when masks are covering so much of the rest of the face. The main issues with eye makeup are contamination and cleaning. It is important you be sure that applicators are clean, products are fresh and non-expired, and hands are clean. If the application of makeup can be done at home in a clean environment and with clean materials, there is no real concern even with the mask. However, re-applying makeup while out in public spaces or with non-clean brushes or applicators and possibly contaminated older material can increase the chances of infection and irritation associated with eye makeup.

### JV

We are seeing a few “buckets” of potential exposure in the professional liability field due to the age of COVID. Healthcare professional liability can cross-reference with occupational health. The buckets include missed diagnosis due to providers being too focused on COVID versus the basics of, for example, glaucoma; missed appointments because patients were initially not coming in due to providers not treating “elective procedures/tests,” for example, a yearly eye exam; as elective procedures/tests were allowed, patients continued to not come in due to fear of COVID exposure. In terms of eye care, it is important to get those yearly exams, tests, and other evaluations. COVID interrupted continuity of care throughout healthcare, eye care is no different.

**Would you share one innovative idea you found helpful in balancing eye health with the challenges posed by the current pandemic?**

### JC

With the ever-increasing use of digital displays and communication devices, we feel the use of basic precautions (20–20–20 rule) is the best option to minimize eye strain and blue light exposure. The research on the use of blue-blocking lenses is non-conclusive, so we cannot recommend them at this time.

### GH

In terms of our practice, I find it equally important to consider the safety of our employees as well as setting the stage for patients to feel safe during the examination. I agree with Mr. Mohrmann in that safety checks throughout the office visit promote safety for all.

### SH

One challenge was reusing goggles or face shields due to a shortage of personal protective equipment (PPE) to protect our healthcare workers. We followed the Centers for Disease Control and Prevention guidelines on reprocessing eye protection (see Table 1).

**Table 1.** Resources to Promote Eye Health in the COVID-19 Pandemic

Centers for Disease Control and Prevention. (2020). *Strategies for optimizing the supply of eye protection*. https://www.cdc.gov/coronavirus/2019-ncov/hcp/ppe-strategy/eye-protection.html Hartford Healthcare. (2020). *Six tips from an ophthalmologist to avoid eye strain*. https://hartfordhealthcare.org/about-us/news-press/newsdetail?articleId=28826&publicid=395 World Health Organization. (2020). *Advice on the use of masks in the context of COVID-19: Interim guidance*. https://apps.who.int/iris/handle/10665/332293

### EH

I suggest the worker be viewed holistically. There are so many adjustments that need to be made that overall health and well-being can be overlooked. Eye health is extremely important; however, this is one element of health that cannot be separated from the overarching goal of well-being. Being aware of the relationship between prolonged sitting and screen time is an example of one adjustment to the remote experience that impacts eye health, productivity, cardiovascular health, and more. It best serves the occupational health professional to recognize that well-being is not simply absence of disease, rather a holistic approach to thriving.

### JV

Blue light glasses, breaks, tracking your screen time, eye exams, and appropriate eye tests are essential to good eye health. Our computers as well as our phones, pads, and other screens can let us know how much screen time we have experienced in a day. There are also built-in breaks or stretch reminders that can be used on our computers. If the pandemic and permanent work from home becomes the new normal, eye health will be affected and we need to stress healthy screen time even more.

## Summary

The COVID-19 pandemic has had an impact on many elements of occupational health and wellness. Sight, a sense often overlooked, has become a topic for discussion. Professionals understand this and so do consumers. For example, an Alcon/Ipsos poll conducted in July 2020, where 1,005 individuals were surveyed, found that those surveyed reported spending more time in front of a screen, nearly half (45%) say that they have experienced their eyes feeling dry as a result, and 60% are concerned about the impact that increased screen time will have on their eyes (Sesetyan & Muschinske, 2020).

Occupational health professionals are in a position to provide training and practical interventions to promote eye health. As the experts explained lighting, the 20–20–20 rule, ergonomically sound furniture, regular professional eye care make a difference.

Limitations to this article include the rapidly evolving nature of the COVID-19 pandemic. In addition, little peer-reviewed evidence exists pertaining to COVID-19, masks, falls, the extent screen time is currently having on the eyes, or the effect the 20–20–20 rule has on the eye in today’s environment. Intervention is largely dependent on historic data, expert opinion, case reports, and consumer polls.
